# Reviewer acknowledgement 2013

**DOI:** 10.1186/1471-2334-14-20

**Published:** 2014-02-07

**Authors:** Philippa K Harris

**Affiliations:** 1 BioMed Central, 236 Gray’s Inn, Road, London, WC1X 8HB, UK

## Abstract

**Contributing reviewers:**

The editors of *BMC Infectious Diseases* would like to thank all our reviewers who have contributed to the journal in Volume 13 (2013).

## 

Andrew Abaasa

Uganda

Kevin Abbott

USA

Fekadu Abebe

Norway

Stephan Aberle

Austria

Sophie Abgrall

France

Asha Abraham

India

Mark Abzug

USA

Amit Achhra

Australia

Kate Adams

UK

Linda Adams

USA

Toidi Adekambi

USA

Oluwatoyin Adeyemi

USA

Yaw Afrane

Kenya

Rakesh Aggarwal

India

William Agger

USA

Selidji Todagbe Agnandji

Gabon

Riris Andono Ahmad

Indonesia

Maximilian C. Aichelburg

Austria

Pedro Aide

Mozambique

Campbell Aitken

Australia

Stella Akinleye

Nigeria

Karolina Akinosoglou

Greece

Mahboob Alam

USA

Samer Alasaad

Spain

Maryam Alavi

Australia

Waleed Alazmi

Kuwait

Guilherme Albertoni

Brazil

Gabriel Alcoba

Switzerland

Elizabeth Alexander

USA

James Alexander

USA

Majdi Al-Hasan

USA

Jean-Pierre Allain

UK

Theresa Allain

Malawi

Victoria Allgar

UK

Aysha Almas

Pakistan

Maribel Almonte

France

Sylvie Alonso

Singapore

Haoues Alout

USA

Claudia Alteri

Italy

Christian Althaus

Switzerland

Danny Altmann

UK

Cosme Alvarado-Esquivel

Mexico

Xavier Alvarez

USA

Andre Amaral

Canada

Amal Amer

USA

Roland Ammann

Switzerland

Neil M Ampel

USA

Suzanne Anderson

Gambia

Monique Andesson

South Africa

David Andresen

Australia

Irene Ang

Hong Kong

Li Wei Ang

Singapore

Wim Ang

Netherlands

Jonathan Angel

Canada

Andrea Angheben

Italy

Spinello Antinori

Italy

Valentin Antoci

USA

Annika Antonsson

Australia

Sami Aoufi Rabih

Spain

Victor Appay

France

Werner Apt

Chile

Imadeldin Aradaib

Sudan

Francisco Eleodoro Arancibia

Chile

Marc Arbyn

Belgium

Carla Renata Arciola

Italy

Carmen Ardanuy

Spain

Joop Arends

Netherlands

Matthew Arentz

USA

Minetaro Arita

Japan

Kaku Armah

USA

Mireia Arnedo Valero

Spain

Catherine Arnold

UK

Valentina Arsic Arsenijevic

Serbia

Victor Asensi

Spain

Usman Ali Ashfaq

Pakistan

Elizabeth Ashley

Thailand

Ojan Assadian

Austria

Katherine Atkins

USA

Alexandra Aubry

France

John Aucott

USA

Etienne Audureau

France

Sara Auld

USA

Anchalee Avihingsanon

Thailand

Muluken Azage

Ethiopia

Andrew Azman

USA

Hideo Baba

Japan

George Babis

Greece

Prasith Baccam

USA

Parisa Badiee

Iran

Henry Baggett

Thailand

Patrizia Bagnarelli

Italy

Kuan-Jen Bai

Taiwan

Mirjam Irene Bakker

Netherlands

Fausto Baldanti

Italy

Vincenzo Baldo

Italy

David Banach

USA

Melanie Bannister-Tyrrell

Australia

Yan-Ping Bao

China

Bridget Barber

Australia

Angela Barbosa

Brazil

Christovam Barcellos

Brazil

Gavin Barlow

UK

Dale Barnard

USA

Lucia Barrera

Argentina

Roberto Barrera

USA

Paola R Barrero

Argentina

Mike Barrett

UK

Jim Barrington

Switzerland

David Barroso

Brazil

Peter Barry

USA

Roos Emilie Barth

Netherlands

Surajit Basak

India

Quique Bassat

Spain

Mathieu Bastard

France

Francisco Bastos

Brazil

David Bates

USA

Matthew Bates

UK

Chris Bauch

Canada

Nicholas Beare

UK

Cecile Bebear

France

Christiane Bebear

France

Angela Bechini

Italy

Ingeborg Becker

Mexico

Karsten Becker

Germany

Marissa Becker

Canada

Branka Bedenic

Croatia

Jerzy Behnke

UK

Sandra Beinhardt

Austria

Ruud Bekkers

Netherlands

Vinicius Belo

Brazil

Shmuel Benenson

Israel

Thomas Benfield

Denmark

Jiri Beran

Czech Republic

Thomas Berg

Germany

Peter Bergman

Sweden

Maria Bergquist

Sweden

Johannes Berkhof

Netherlands

Nicole Bernard

Canada

Alexandra Bernards

Netherlands

Andrea Bernini

Italy

Antonio Bertoletti

Singapore

Julie Bertrand

UK

Victoria Best

USA

Tom Bewick

UK

Getenet Beyene

Ethiopia

Samit Bhattacharyya

USA

Taufiqur Bhuiyan

Bangladesh

Asaf Biber

Israel

Erwin Biecker

Germany

Robin Biellik

UK

Cedric Bien

USA

Matthew Biggerstaff

USA

Jade Bilardi

Australia

Kristine Bisgard

USA

Cyrille Bisseye

Burkina Faso

Jyotirmay Biswas

India

Jorge Blanco

Spain

José-Ramón Blanco

Spain

Maaike Bleeker

Netherlands

Thomas Bock

Germany

Linda Bockenstedt

USA

Barbara Body

USA

Nicholas Boire

USA

Vicente Boix

Spain

Pierre-Edouard Bollaert

France

Paolo Bonanni

Italy

Maryline Bonnet

Switzerland

Laura Bonnett

UK

Ken Boorom

USA

Jikke Bootsma

Netherlands

Martin Bootsma

Netherlands

Hanneke Borgdorff

Netherlands

Steffen Borrmann

Germany

Karina Bortoluci

Brazil

Steven Bosinger

USA

Aaron Bossler

USA

John Bosso

USA

Kocjan Bostjan

Slovenia

Tim Boswell

UK

Graham Bothamley

UK

Alex Bottle

UK

Marielle Karine Bouyou-Akotet

Gabon

Scott Bowden

Australia

Anders Boyd

France

Marek Brabec

Czech Republic

Daniel Bradshaw

Australia

Cynthia Braga

Brazil

Lucia Braga

Brazil

Paula Braitstein

USA

Maria Cristina Brandileone

Brazil

Sabine Brandt

Austria

Eimear Brannigan

UK

Patricia Brasil

Brazil

Eyal Braun

Israel

Stéphane Bretagne

France

Michael Brimacombe

USA

Cristiana Brito

Brazil

B.D. Lidewij Broekhuizen

Netherlands

Shobha Broor

India

Florence Brossier

France

Julia Brotherton

Australia

Peter Brotherton

UK

Matthijs Brouwer

Netherlands

Derek Brown

UK

Kevin Brown

UK

Nele Brusselaers

Sweden

Brita Bruun

Denmark

Johan Nikolai Bruun

Norway

Bryce Buddle

New Zealand

Christine Budke

USA

Guillermo Bugedo

Chile

Wallace Bulimo

Kenya

Ann Burchell

Canada

Timothy Burgess

USA

Gheorghe Burnei

Romania

Cara Burns

USA

Sarah Burr

Gambia

Elena Burroni

Italy

Luca Busani

Italy

Anne Buve

Belgium

Josephine Bwogi

Uganda

André Cabié

Martinique

Edward Cachay

USA

Tommaso Cai

Italy

Nadege Caillere

France

Morena Caira

Italy

Andrea Calcagno

Italy

Annapaola Callegaro

Italy

Steve Calvano

USA

Carlos Camargo

Brazil

Bernard Camins

USA

Teresa Camou

Uruguay

Maiza Campos Ponce

Netherlands

Ellen Caniglia

USA

Bin Cao

China

Maria Grazia Capretti

Italy

Alex Carballo-Dieguez

USA

Celso Cardoso

Brazil

Divina Cardoso

Brazil

Philip Carling

USA

Francisco Javier Carod Artal

UK

Verena I. Carrara

Thailand

Luis Roman Carrasco

Singapore

Patrizia Carrieri

France

Lauren Carrington

Vietnam

Enitan Carrol

UK

E Jane Carter

USA

Anna Casey

UK

Jennifer Cashdollar

USA

Beatrice Casini

Italy

Claudio Casoli

Italy

Jorge Casseb

Brazil

Antonio Cassone

Italy

Elio Castagnola

Italy

Mariana Castanheira

USA

Francesco Castelli

Italy

Barbara Castelnuovo

Uganda

Paolo Castiglia

Italy

Jose Castro

USA

Chiara Cattaneo

Italy

Giovanni Cattoli

Italy

Simon Cauchemez

France

Carlos Eugênio Cavasini

Brazil

Dayaraj Cecilia

India

Benedetto Maurizio Celesia

Italy

Tomasa Centella

Spain

Thomas Cesario

USA

Paola Chabay

Argentina

Magali Chabé

France

Wanpen Chaicumpa

Thailand

Nathorn Chaiyakunapruk

Thailand

Pradip Chakraborti

India

Runu Chakravarty

India

James Chalmers

UK

Jasper Chan

Hong Kong

Paul Chan

Hong Kong

Wilson Chan

Canada

Yoke-Fun Chan

Malaysia

Manish Chandey

India

Partha Chandra

USA

Nagarathna Chandrashekar

India

Ram Chandyo

Nepal

Cheejen Chang

Taiwan

Feng-Yee Chang

Taiwan

Shan-Chwen Chang

Taiwan

Ting-Tsung Chang

Taiwan

Wen-Neng Chang

Taiwan

Narisara Chantratita

Thailand

Jacques Derek Charlwood

UK

Kalyan Chavda

USA

Novel N Chegou

South Africa

Chih-Jung Chen

Taiwan

Fei Chen

USA

Feng-Jui Chen

Taiwan

Liang Chen

USA

Luke Chen

USA

Ming Chen

China

Po-Yen Chen

Taiwan

Yin-Bun Cheung

Singapore

Stephane Chevaliez

France

Christophe Chevillard

France

Rong-Nan Chien

Taiwan

Shingo Chihara

USA

Violet Chihota

South Africa

Natsayi Zanile Chimbindi

South Africa

Ng Lee Ching

Singapore

Brian Chiu

Canada

Charles Chiu

USA

Cheng-Hsun Chiu

Taiwan

Yoon Hong Choi

UK

Young Ki Choi

South Korea

Chia Yin Chong

Singapore

Virasakdi Chongsuvivatwong

Thailand

Mouhamadou Chouaibou

Cote d'Ivoire

Angela Chow

Singapore

Gerardo Chowell

USA

Nicole Christian

Jamaica

Kuaban Christopher

Cameroon

Yiu Wai Chu

Hong Kong

Wan-Long Chuang

Taiwan

Ampaiwan Chuansumrit

Thailand

Gavin John Churchyard

South Africa

Massimo Ciccozzi

Italy

Luka Cicin-Sain

Germany

Iza Ciglenecki

Switzerland

Daniela M Cirillo

Italy

Jose M. Cisneros

Spain

C. Graham Clark

UK

Jesse Clark

USA

Stuart Clarke

UK

Christine Elisabeth Clavel

France

Philippe Clevenbergh

Myanmar

Gary Clifford

France

Nathan Clumeck

Belgium

Nicol Coetzee

UK

Adam Cohen

South Africa

Justin Cohen

USA

Ted Cohen

USA

Vittoria Colizza

France

Beth-Ann Coller

USA

Arnaldo Lopes Colombo

Brazil

Philippe Colson

France

Christophe Combet

France

Barry Combs

Australia

Anali Conesa-Botella

Belgium

Ulisses Confalonieri

Brazil

Rafael Contreras Galindo

USA

Fiona Cooke

UK

Ben Cooper

Thailand

Lawrence Copelovitch

USA

Ross Coppel

Australia

Nicola Coppola

Italy

Debora Coraca-Huber

Austria

Anne Cori

UK

Alyssa Cornall

Australia

Anna-Maria Costa

Australia

Jean Tenena Coulibaly

Cote d'Ivoire

Wendel Coura-Vital

Brazil

Benjamin Cowling

Hong Kong

Helen Cox

Australia

Michael Craig

USA

Jakob Peter Cramer

Germany

Lisa Cranmer

USA

Dana Crawford

USA

Alan Cross

USA

Amy Crowe

Australia

Pablo Cruces

Chile

Heather Cubie

UK

Manuel Cuenca-Estrella

Spain

Maria De Lourdes Cunha

Brazil

Marian Currie

Australia

Scott Curry

USA

Jeffery Cutter

Singapore

Christopher Czaja

USA

Gabriela Da Silva

Portugal

Maria Alice Da Silva Telles

Brazil

Alda Maria Da-Cruz

Brazil

Ron Dagan

Israel

Gunnar Dahlen

Sweden

Chia-Yen Dai

Taiwan

Koustuv Dalal

Sweden

Charles Daley

USA

Harry Dalton

UK

David Dance

Laos

Collet Dandara

South Africa

Nick Daneman

Canada

Alison Dann

Australia

Lara Danziger-Isakov

USA

Toni Darville

USA

Sibnarayan Datta

India

Siddhartha Datta

India

Michael David

USA

Sascha David

Germany

Donald Davidson

UK

Nathalie Davoust

France

George Dawson

USA

Carolyn Day

Australia

Anuradha De

India

Bertille De Barbeyrac

France

Jessica De Beer

Netherlands

Anna De Breij

Netherlands

Ivano De Filippis

USA

Cornelis De Haan

Netherlands

Cornelis Pc De Jager

Netherlands

Birgitta De Jong

Sweden

R.C.J. De Jonge

Netherlands

Isabel De Kantor

Argentina

Xavier De Lamballerie

France

Andrea De Luca

Italy

Andrea De Maria

Italy

Theo M. De Reijke

Netherlands

Andrés De Roux

Germany

Silvia De Sanjose

Spain

An De Sutter

Belgium

Henriette De Valk

France

Philippe De Wals

Canada

Stephane De Wit

Belgium

Keith Dear

Australia

Anne Deckert

Canada

Jean-Winoc Decousser

France

Huang Dedong

China

Shelley Deeks

Canada

Abraham Degarege

Ethiopia

Julia Del Amo

Spain

Valerio Del Bono

Italy

Jose Del Campo

Spain

Fernando Del Fiol

Brazil

Patricia Del Portillo

Colombia

Sara Del Valle

USA

Frank Deleo

USA

Giovanni Delogu

Italy

Vittorio Demicheli

Italy

Meaza Demissie

Ethiopia

Casper Den Heijer

Netherlands

Pieter Depuydt

Belgium

Kebede Deribe

Ethiopia

Amare Deribew

Ethiopia

David Deshazer

USA

Abhishek Deshpande

USA

Lalitagauri Deshpande

USA

Daniel Desimone

USA

Gabriella D'Ettorre

Italy

Floyd Dewhirst

USA

Sohini Dey

India

Amreeta Dhanoa

Malaysia

Keertan Dheda

South Africa

Ramesh Dhiman

India

Maria Silvia Di Genaro

Argentina

Cicero Dias

Brazil

Fredi Alexander Diaz-Quijano

Colombia

Roland Diel

Germany

Trinidad Dierssen Sotos

Spain

Javier Diez-Domingo

Spain

Peter John Diggle

UK

Lenie Dijkshoorn

Netherlands

Jo-Anne R Dillon

Canada

Isabelle Dimier-Poisson

France

George Dimopoulos

Greece

Trung Dinh The

Vietnam

Zorana Djordjevic

Serbia

Olgica Djurkovic-Djakovic

Serbia

Hana Dobrovolny

USA

Hazel Dockrell

UK

Pete Dodd

UK

Mehmet Doganay

Turkey

Tom Doherty

UK

Yohei Doi

USA

Eugen Domann

Germany

Cristina Domingo

Spain

Pere Domingo

Spain

Regina Domingues

Brazil

Àngela Domínguez

Spain

Francesco Donato

Italy

Blaise Dondji

USA

Tao Dong

UK

Susan Dorman

USA

Horng-Yunn Dou

Taiwan

Richard Douglas

New Zealand

Paul Drain

USA

Alison Drake

USA

Erik Dubberke

USA

Ann-Sofi Duberg

Sweden

Kwabena Duedu

UK

Radboud Duintjer Tebbens

USA

Chris Duncan

UK

Paolo Durando

Italy

Malcolm Duthie

USA

Richard Dwyer

Sweden

Ravi Dyavar

USA

D Ted Eastlund

USA

Jose Echevarria

Spain

Philip Eckhoff

USA

Charles Edwards

Barbados

David Edwards

UK

Kathryn Edwards

USA

Matthias Egger

Switzerland

Boris P Ehrenstein

Germany

Michael Eisenhut

UK

Anna Eis-Huebinger

Germany

Zelal Ekinci

Turkey

Koumavi Didier Ekouevi

Cote d'Ivoire

Chukwuma Ekpebegh

South Africa

Uwem Ekpo

Nigeria

Gary Eltringham

UK

George Eluwa

Nigeria

Henk W Elzevier

Netherlands

Chijioke Emenike

Malaysia

Ochola Emmanuel

Uganda

Joanna Empel

Poland

Javier Ena

Spain

Tomoyuki Endo

Japan

Eric Engels

USA

Peter English

UK

Hakan Erdem

Turkey

Britt-Marie Eriksson

Sweden

Josep M. Escribá

Spain

Vincent Escuyer

USA

Semih Esin

Italy

Clara Espitia

Mexico

Susanna Esposito

Italy

Jaime Esteban

Spain

Angel Estella

Spain

Vicente Estrada

Spain

Denise Evans

South Africa

Sayeh Ezzikouri

Morocco

Massimiliano Fabbiani

Italy

Christopher Fairley

Australia

Roger Faix

USA

Chiara Falciani

Italy

Joseph Falkinham

USA

Liam Fanning

Ireland

Tamer Farag

USA

Darise Farris

USA

Shohreh Farshad

Iran

Daniel Faurholt-Jepsen

Denmark

Saul Faust

UK

Warren Fawley

UK

Edward Feller

USA

J. Christopher Fenno

USA

Alan Fenwick

UK

Nuria Fernández-Hidalgo

Spain

Mario Fernández-Ruiz

Spain

Sumadhya Fernando

Sri Lanka

Joseli Ferreira

Brazil

Michael Ferris

USA

Tristan Ferry

France

James Fielding

Australia

Richard Fielding

Hong Kong

Kenneth Fife

USA

Antonietta Filia

Italy

Volker Fingerle

Germany

Katja Fink

Singapore

Dale Fisher

Singapore

Elmira Flem

Norway

Douglas Fleming

UK

Fiona Fleming

UK

Steffen Flessa

Germany

Emanuele Focà

Italy

Andrew Fogarty

UK

Marcelo Fonseca

Brazil

Simone Fonseca

Brazil

Emmanuel Forestier

France

Pia Forsberg

Sweden

Jeffrey Foster

USA

Vasileios Fotopoulos

Greece

Freya Fowkes

Australia

Elisabetta Franco

Italy

Carlos Franco-Pparedes

USA

John Frean

South Africa

Maria Fredriksson-Ahomaa

Finland

Christopher Frei

USA

Neil French

Malawi

Dineke Frentz

Netherlands

Gerald Friedland

USA

Alexander W. Friedrich

Netherlands

Ingrid Friesema

Netherlands

Nina Friis-Møller

Denmark

Anne Frosch

USA

Jennifer Furin

USA

Wieslaw Furmaga

USA

Jon Furuno

USA

Francesco Maria Fusco

Italy

Lucia Gabriele

Italy

Terhes Gabriella

Hungary

Giovanni Gabutti

Italy

Naomi Gadsby

UK

Sebastien Gagneux

Switzerland

Angel S. Galabov

Bulgaria

Renee Galloway

USA

Yunn-Hwen Gan

Singapore

Anuradha Ganesan

USA

Maria Garces-Sanchez

Spain

Davinder Garcha

UK

Federico Garcia

Spain

Joao Luis Garcia

Brazil

Josefina Garcia

Peru

Maria Luz Garcia-Garcia

Spain

Bradley Gardiner

Australia

Adrian Gardner

Kenya

Shea Gardner

USA

Franco Gargiulo

Italy

Marisa Gariglio

Italy

Paul Garner

UK

Roberto Gasparini

Italy

Jose M Gatell

Spain

Nikolaos Gatselis

Greece

Andrea Gazzinelli

Brazil

Dongliang Ge

USA

Ruiguang Ge

China

Berhanu Gebremeskel

USA

Marila Gennaro

USA

Maha Ghanem

Egypt

Giovanni Gherardi

Italy

Souvik Ghosh

Japan

Susanta Ghosh

India

Reza Ghotaslou

Iran

Luisa Giaccone

Italy

Nicola Gianotti

Italy

Heather Gidding

Australia

Norberto Giglio

Argentina

Angel Gil

Spain

Brent Gilpin

New Zealand

Ruth Gil-Prieto

Spain

Raphaële Girard

France

Enrico Girardi

Italy

Effrossyni Gkrania - Klotsas

UK

Aharona Glatman-Freedman

USA

Erik Glocker

Germany

Jacques Godfroid

Norway

Darla Goeres

USA

Walter Goessler

Austria

Charalambos Gogos

Greece

Namraj Goire

Australia

Takashi Gojobori

Japan

David Goldberg

UK

Jane Goller

Australia

Selma De Andrade Gomes

Brazil

Yara Miranda Gomes

Brazil

Beatriz Lucia Gomez Giraldo

Colombia

Stevan Gonzalez

USA

Chulananda Goonasekera

UK

Nicola Gordon

UK

Gaetano Gorgone

Italy

Peter Goroncy-Bermes

Germany

Roly Gosling

USA

Neela Goswami

USA

Pierre Goussard

South Africa

Jérôme Gouttenoire

Switzerland

Michele Gouvea

Brazil

Brian Graham

USA

Susan Graham

USA

Francesco Grandesso

France

Denis Grandgirard

Switzerland

Kathie Grant

UK

Nicholas Graves

Australia

Patricia Graves

Australia

Stephen Graves

Australia

Jeremy Gray

Ireland

Magdalena Grce

Croatia

Jason Grebely

Australia

Ezekiel Green

South Africa

Christopher Gregory

USA

Daniel Gregson

Canada

Allen Griffin

USA

David Griffith

USA

Pierfrancesco Grima

Italy

Deanna Grimes

USA

Martin Peter Grobusch

Netherlands

Andreas H. Groll

Germany

Uwe Gross

UK

Pawel Grzesiowski

Poland

Peng Guan

China

Yi Guan

China

Giovanni Guaraldi

Italy

Carlota Gudiol

Spain

Marguerite Guiguet

France

Thaís Guimarães

Brazil

Jesus Guinea

Spain

Javier Guitian

UK

Sunethra Gunasena

Sri Lanka

Murat Gunaydin

Turkey

M. Cristina Gutierrez

France

Ramiro Gutierrez

USA

Lee Ha Youn

South Korea

Eric Haarman

Netherlands

David Haas

USA

Mustafa Hacimustafaoglu

Turkey

Michele Hacker

USA

Azar Hadadi

Iran

Ivanka Hadji-Petrusheva Meloska

Macedonia

Chadi Hage

USA

Behzad Hajarizadeh

Australia

John Halperin

USA

Scott Halstead

USA

Sari Hämäläinen

Finland

Richard Hamilton

USA

Margaret Hammerschlag

USA

Sarah Hammond

USA

Klaus Hamprecht

Germany

Eun-Taek Han

South Korea

Jennifer Han

USA

David Hanna

USA

Stypulkowska Hanna

Poland

Colleen Hanrahan

USA

Stephan Harbarth

Switzerland

Richard Harding

UK

Kim Hare

Australia

Megan Hargreaves

Australia

Julie Harris

USA

Habsah Hasan

Malaysia

Nur Hasan

USA

Rodrigo Hasbun

USA

Angelos Hatzakis

Greece

Thomas Haupt

USA

Jennifer Havens

USA

Nancy Havill

USA

Claudia Hawkins

USA

Thomas Hawn

USA

Kayoko Hayakawa

Japan

Tetsuya Hayashi

Japan

Mary Hayden

USA

Ming-Liang He

China

Qiushui He

Finland

Qun He

China

Sarah Head

UK

Craig Hedberg

USA

Bethany L Hedt-Gauthier

USA

Janneke Heijne

Switzerland

Yuji Heike

Japan

Markus Hell

Austria

Elizabeth Henderson

Canada

Ilse Hendriksen

Netherlands

Moon Her

South Korea

Jean-Michel Heraud

Madagascar

Ottmar Herchenröder

Germany

Thomas Herchline

USA

Rodrigo T Hernandes

Bouvet Island

Brenda Hernandez

USA

Rigoberto Hernandez-Castro

Mexico

Erik Hewlett

USA

Paul Heyman

Belgium

Anke Hildebrandt

Germany

Andrew Hill

New Zealand

Philip Hill

New Zealand

Doris Hillemann

Germany

Sven Gudmund Hinderaker

Norway

Musa Hindiyeh

Israel

Naoki Hiramatsu

Japan

Pengiran Hishamuddin

Singapore

Laurent Hocqueloux

France

Martin Hoenigl

Austria

Daniel Hoft

USA

Peter Hogan

Australia

Elizabeth Hohmnn

USA

Benjamin Holder

USA

Natasha Holmes

Australia

Michael Hombach

Switzerland

Chun-Yip Hon

Canada

Kam-Lun Ellis Hon

Hong Kong

Michael Horberg

USA

Nobuyuki Horita

Japan

David Horn

USA

Salih Hosoglu

Turkey

Duane Hospenthal

USA

Rein M G J Houben

UK

Eric Houpt

USA

Benjamin Howden

Australia

Yu-Hsiang Hsieh

USA

Chao Wei Hsu

Taiwan

Ching-Sheng Hsu

Taiwan

Li-Yang Hsu

Singapore

Chun Huang

China

De-Sheng Huang

China

Xiaozhe Huang

USA

Yhu-Chering Huang

Taiwan

Kelly Huber

USA

Anke Huckriede

Netherlands

Stéphane Hué

UK

Linda Hueston

Australia

Edward Hughes

UK

Christopher Hui

Hong Kong

Veijo Hukkanen

Finland

Ivan Fn Hung

Hong Kong

Elizabeth Hunsperger

Puerto Rico

Gillian Hunt

South Africa

Shirish Huprikar

USA

Mina Hur

Kyrgyzstan

James Hurley

Australia

Aeron Hurt

Australia

Nguyen Tien Huy

Japan

Christos Iavazzo

Greece

Lailanor Ibrahim

Malaysia

M. Idrees

Pakistan

Valerio Iebba

Italy

Yoshifumi Imamura

Japan

Arkaitz Imaz

Spain

Patrik Inderbitzin

USA

Anna Maria Iorio

Italy

Margaret Ip

Hong Kong

Jose Irazuzta

USA

Kathleen Irwin

USA

David Isaacs

Australia

Naoto Ishimaru

Japan

Nahed Ismail

USA

Miren Iturriza

UK

Pavel Ivanov

USA

Katsuomi Iwakura

Japan

Kentaro Iwata

Japan

Jacques Izard

USA

Jacques Izopet

France

Hidemasa Izumiya

Japan

Slawomir Jablonski

Poland

Debra Jackson

South Africa

Shabbar Jaffar

UK

Shah Jahan

Pakistan

Seema Jain

USA

Michel Janier

France

Vincent Jarlier

France

Lina Jaruseviciene

Lithuania

Marjan Javanbakht

USA

Seyed Mohammad Jazayeri

Iran

Jo Jefferies

UK

Grant Jenkin

Australia

Henrik Jensen

Denmark

Jens-Ulrik Jensen

Denmark

Joseph Jeong

South Korea

Cameron Jeremiah

Australia

Minjun Ji

China

Qingmei Jia

USA

Wei Jiang

China

Yiling Jiang

UK

Judy Natalia Jiménez

Colombia

Maria Angeles Jimenez-Sousa

Spain

Fengyi Jin

Australia

Qi Jin

China

Mark Jit

UK

Barbara W. Johnson

USA

Cheryl Jones

Australia

Noyal Joseph

India

Sarah Jourdain

Belgium

Carlos Juan Nicolau

Spain

Lia Monica Junie

Romania

Anamarija Jurcev Savicevic

Croatia

Amy Justice

USA

Auni Juutilainen

Finland

Vidyalakshmi K

India

Achim Kaasch

Germany

Masanori Kai

Japan

Siripen Kalayanarooj

Thailand

Andre Kalil

USA

Teemu Kallonen

Finland

Andrew Kambugu

Uganda

Amine Kamen

Canada

Regianne Umeko Kamiya

Brazil

Guenter Kampf

Germany

Cheol-In Kang

South Korea

G Kang

India

Young Ae Kang

South Korea

Alan Kaplan

Canada

Gilla Kaplan

USA

Drosos Karageorgopoulos

Greece

Nabil Karah

Norway

Erik Karlsson

USA

Shannon Kasperbauer

UK

Desta Kassa

Ethiopia

Achilles Katamba

Uganda

Hormuzd Katki

USA

Midori Kato-Maeda

USA

Elisabete Kawakami

Brazil

Takashi Kawamura

Japan

Oya Kayacan

Turkey

Michael Kays

USA

Lawrence Ndekeleni Kazembe

Malawi

Karen Keddy

South Africa

Theodoros Kelesidis

USA

Brian Kendall

USA

Dervla Kenna

UK

Opio Kenneth

Uganda

Chris Kenyon

South Africa

Can Kesmir

Netherlands

Alison Kesson

Australia

Shravan Kethireddy

USA

Daniel Kett

USA

Olen Kew

USA

Azra Khanum

Pakistan

Ali Khawaja

Pakistan

Thana Khawcharoenporn

USA

Prasanna Khot

USA

Yury Khudyakov

USA

Benson Kidenya

Tanzania

Jimmy Kihara

Kenya

Dalokay Kilic

Turkey

Gerry Killeen

Tanzania

Arthur Kim

USA

H. Hyune-Ju Kim

USA

Kouji Kimura

Japan

Julie Kingery

USA

Naoko Kinoshita-Katahashi

Japan

Janni Kinsler

USA

Patricia Kipnis

USA

Louis Kirchhoff

USA

Martyn Kirk

Australia

Lea-Ann Kirkham

Australia

Theo Kirkland

USA

Herbert Kiss

Austria

Istvan Kiss

Hungary

Patty Kissinger

USA

Alan Kitchen

UK

Leera Kittigul

Thailand

Nicholas Kiulia

Kenya

Anne Kjemtrup

USA

Nichole Klatt

USA

Silke R. Klee

Germany

Matthias Klein

Germany

Terry Klein

USA

Helmut Kloos

USA

Hilde Kløvstad

Norway

Stephen Knabel

USA

Nobumichi Kobayashi

Japan

Tamaki Kobayashi

USA

Bekir Kocazeybek

Turkey

Renate Koenig

Germany

John Koethe

USA

Kwee Choy Koh

Malaysia

Tse Hsien Koh

Singapore

Won-Jung Koh

South Korea

Satoshi Koike

Japan

Nobuo Koizumi

Japan

Bill Kojola

USA

Martin Kolditz

Germany

Marin Kollef

USA

Haruki Komatsu

Japan

Onn Min Kon

UK

Petros Kopterides

Greece

Titia Kortbeek

Netherlands

Claudio Köser

UK

Markus Kostrzewa

Germany

Roger Kouyos

Switzerland

Mel Krajden

Canada

Axel Kramer

Germany

Anna Kramvis

South Africa

Rafal Krenke

Poland

Marco Aurelio Krieger

Brazil

John Kriesel

USA

Andreas Krueger

Germany

Ralf Krumkamp

Germany

Deanna Kruszon-Moran

USA

Chia-Chi Ku

Taiwan

Ernest Kuchar

Poland

Krystle Kuhs

USA

Senanayake Kularatne

Sri Lanka

Sangeeta Kulkarni

India

Nilgün Kültürsay

Turkey

Yadunanda Kumar

USA

Newton Kumwenda

Malawi

Kin On Kwok

Hong Kong

Bernard La Scola

France

Giuseppe La Torre

Italy

Martin Laass

Germany

Alain Labrique

USA

Olivier Lada

France

Dora Lam-Himlin

USA

Patrick Lammie

USA

Mohammed Lamorde

Uganda

Christina Lancioni

USA

Paolo Landini

Italy

Christian Lange

Germany

Joanne Langley

Canada

Liu Langxia

China

Rafael Laniado-Laborin

Mexico

Mareike Lankeit

Germany

Daniele Lantagne

USA

Paul Lantos

USA

Malinee Laopaiboon

Thailand

Giuseppe Lapadula

Italy

Sarah Larney

Australia

Regina Larocque

USA

Bernard Larouze

France

Margaret Lartey

Ghana

Christine Lascols

USA

Kayla Laserson

USA

Anna Lass

Poland

Cornelia Lass-Floerl

Austria

Eric Lau

Hong Kong

Tsai-Ling Lauderdale

Taiwan

Kevin Laupland

Canada

Dortet Laurent

France

Jean-Philippe Lavigne

France

Matthew Law

Australia

Stephen Lawn

South Africa

Stephen Lawn

UK

Marzia Lazzerini

Italy

Thuy Le

Vietnam

Karin Leder

Australia

Catherine Lee

USA

Grace Lee

USA

Ming-Hsun Lee

Taiwan

Ping-Ing Lee

Taiwan

Sunmi Lee

USA

Vernon Lee

Singapore

William Lee

USA

Surbhi Leekha

USA

David Leiby

USA

Jami Leichliter

USA

Tatjana Lejko Zupanc

Slovenia

Rose Leke

Cameroon

Bertrand Lell

Germany

Sebastain Lemmen

Germany

Martina Lengerova

Czech Republic

Erica Leoni

Italy

Agnès Lepoutre

France

Dragan Lepur

Croatia

Geert Leroux-Roels

Belgium

Olivier Leroy

France

Richard Lessells

South Africa

Laurent Letrilliart

France

Elke Leuridan

Belgium

Anna Levin

Brazil

Vivian Levy

USA

David Lewis

South Africa

Dyani Lewis

Australia

Paul Lewis

USA

Maarten Leyssen

Belgium

François L'Hériteau

France

Linlin Li

USA

Tianming Li

China

Yun-Fan Liaw

Taiwan

Daniel Libraty

USA

Alan Lifson

USA

Troels Lillebaek

Denmark

Patrick Lillie

UK

Tow-Keang Lim

Singapore

Yu-Ting Lin

Taiwan

Nilton Lincopan

Brazil

Jeffrey Linder

USA

Magnus Lindh

Sweden

Mark Lindsley

USA

Jan Lindstrom

UK

Daphne Ling

Canada

Li Ling

China

Melissa Li-Ng

USA

Gabor Linthorst

Netherlands

Delong Liu

USA

Feng Liu

USA

Pengbo Liu

USA

Tao Liu

USA

Zheng Liu

China

Carl Llor

Spain

Hsiu-Jung Lo

Taiwan

Shawn Lockhart

USA

Sara Lodi

Spain

Susan Logan

Canada

Brian Long

USA

Guido Lopes Dos Santos Santiago

Belgium

Mariola Lopez

Spain

Thomas Löscher

Germany

Richard Louie

USA

Nacer Lounis

Belgium

Charito Love

USA

Cheng-Hsien Lu

Taiwan

Hongzhou Lu

China

Jean-Christophe Lucet

France

Vivian Luchsinger

Chile

Alison Ludwig

USA

Tricia Luhn

USA

Alexander Lukashev

UK

Aroonlug Lulitanond

Thailand

Francoise Lunel Fabiani

France

David Lung

Hong Kong

Jun Luo

China

Irja Lutsar

Estonia

Roberto Luzzati

Italy

David Chien Lye

Singapore

Donald Lyon

UK

Xuejun Ma

China

Gary Maartens

South Africa

Musawenkosi Mabaso

South Africa

Neil Macdonald

UK

Alex Mackay

UK

Alexander Mackinnon

USA

Margaret Mackinnon

Kenya

Peter Macpherson

UK

Rebecca Madan

USA

Giordano Madeddu

Italy

Jeroen Maertzdorf

Germany

Markus Maeurer

Sweden

Dhammika Magana-Arachchi

Sri Lanka

Fabio Magurano

Italy

Namita Mahapatra

India

Dermot Maher

Uganda

Rehab Mahmoud Abd El-Baky

Egypt

Hassan Mahomed

South Africa

Marcelo Maia

Brazil

Martin Maiden

UK

Jean-Yves Maillard

UK

Matthias Maiwald

Singapore

Gathsaurie Malavige

Sri Lanka

Karl Malcolm

UK

Bharti Malhotra

India

Nancy Malla

India

Patrick Mallia

UK

Joseph Malone

USA

Denis Malvy

France

Toshie Manabe

Japan

Sundhiya Mandalia

UK

Piero Manfredi

Italy

Reeta Mani

India

Farrin Manian

USA

Balaji Manicassamy

USA

Annette Mankertz

Germany

Barbara Mann

USA

Jennifer Manne

USA

Weerawat Manosuthi

Thailand

Oriol Manuel

Switzerland

Jean-Claude Manuguerra

France

Limin Mao

Australia

Borderi Marco

Italy

Luciano Mariani

Italy

J. Antonio Marin-Neto

Brazil

Sophia Markantonis

Greece

Nikolaos Markou

Greece

Rumyana Markovska

Bulgaria

Michael Marks

UK

Alexandre Marra

Brazil

Theodore Marras

Canada

Jane Marsh

USA

Mariela Martínez Gómez

Brazil

Celina Martelli

Brazil

Thomas Marth

Germany

Hanspeter Marti

Switzerland

David H. Martin

USA

Stanley Martin

USA

Belen Martin Agueda

Spain

Miguel Martinacultad De Medicina

Spain

Adrian Martineau

UK

Esteban Martinez

Spain

María Angélica Martínez

Chile

Regina Martins

Brazil

Neil Martinson

South Africa

Heike Martiny

Germany

Marinko Marusic

Croatia

Takaya Maruyama

Japan

Charis Marwick

UK

Florian Marx

Germany

Sundari Mase

USA

Aleksander Masny

Poland

Ruth Massey

UK

Marguerite Massinga Loembé

Gabon

Naoki Masuda

Japan

Catharina Matheï

Belgium

Barun Mathema

USA

Barun Mathema

USA

Kousaku Matsubara

Japan

Alberto Matteelli

Italy

Petri Mattila

Finland

Isabel Mauricio

Portugal

Ryan Maves

USA

Michail Mavros

USA

Simnikiwe Mayaphi

South Africa

Bryan Mayer

USA

Astrid Mayr

Austria

Lawrence Mbuagbaw

Cameroon

Gary Mcauliffe

New Zealand

James Mccaw

Australia

Michael Mcconnell

Spain

Rose Mcgready

Thailand

Edwin David Mcintosh

UK

James Mckinnell

USA

David Mckinsey

USA

Megan Mclaughlin

China

Ruth Mcnerney

UK

Takafira Mduluza

Zimbabwe

Bonnie Meatherall

Canada

Ingmar Mederacke

USA

Vikram Mehraj

Pakistan

Peter Melby

USA

Szilvia Melegh

Hungary

Alexander Mellmann

Germany

Rodrigo Mendes

USA

Francesco Menichetti

Italy

Rob Menzies

Australia

Dominik Mertz

Canada

Gokhan Metan

Turkey

Mark Metersky

USA

Konradin Metze

Brazil

Pascal Meylan

Switzerland

Fred Mhalu

Tanzania

Jaap M. Middeldorp

Netherlands

Janet Midega

Kenya

Adane Mihret

Ethiopia

Makoto Miki

Japan

Mark Mikkelsen

USA

Rafael T Mikolajczyk

Germany

Malgorzata Mikulska

Italy

L.G. Milagres

Brazil

Eric Mintz

USA

Kiran Mir

USA

Jean Paul Mira

France

Audrey Mirand

France

Dan Miron

Israel

Satoshi Mitarai

Japan

Beatriz Mitrzyk

USA

Masaki Miyake

Japan

Spiros Miyakis

Greece

Naoyuki Miyashita

Japan

Abeer Moanna

USA

Sarah Moberley

Australia

Rebekah Moehring

USA

Trine Mogensen

Denmark

Igor Mokrousov

Russia

Dinesh Mondal

Bangladesh

Susana Monge

Spain

Emmanuel Montassier

France

Patrick Moonan

USA

Catherine Moore

UK

Enrique Morett

Mexico

Daniel Morgan

USA

Jane Morgan

New Zealand

Florent Morio

France

Maria Luisa Moro

Italy

Susan Morpeth

Kenya

Martina Morris

USA

Brenda Morrow

South Africa

Giulia Morsica

Italy

Joel Mossong

South Africa

Roger Moyou-Somo

Cameroon

Stephen Mshana

Tanzania

Sekesai Mtapuri-Zinyowera

Zimbabwe

Lapo Mughini Gras

Netherlands

Khitam Muhsen

Israel

Matthew Muller

Canada

Andargachew Mulu

Ethiopia

Kenneth Munge

Kenya

Flor Munoz

USA

Laura Munoz

Spain

Carmen Munoz-Almagro

Spain

David Murdoch

New Zealand

David Murdoch

USA

Manoj Murhekar

India

Philip Murphy

Ireland

Srinivas Murthy

Canada

Steady Mushayabasa

Zimbabwe

Carlos Muskus

Colombia

Kim Musser

USA

Didier Musso

French Polynesia

Tehmina Mustafa

Norway

Jennie Musto

Australia

Ingomar Mutz

Austria

Mkaya Mwamburi

USA

Joseph Mwangangi

Kenya

Ling Na

Canada

Shobha Nadagir

India

Payam Nahid

USA

Reshma Naik

USA

Damalie Nakanjako

Uganda

Prashant Nasa

India

Ana Lucia Nascimento

Brazil

Marcelle Nascimento

USA

Nairl Nassar

USA

Kalimuthusamy Natarajaseenivasan

India

Shevanthi Nayagam

UK

Seema Nayak

USA

Andrew Naylor

USA

Momar Ndao

Canada

Nicaise Ndembi

Nigeria

Cyrille Ndo

Cameroon

Keith Neal

UK

Barry Neish

UK

Kenrad Nelson

USA

Lisa Nelson

Switzerland

Heinrich Neubauer

Germany

Jacqueline Neuhaus

USA

Mah-Lee Ng

Singapore

John Nicholls

Hong Kong

Audrey Nicholson

UK

Mark Nicol

South Africa

Matthias Niedrig

Germany

Stefan Niemann

Germany

Vassilios Nikolaou

Greece

Hans Helmut Niller

Germany

Nobuyuki Nishikiori

Philippines

Hidekazu Nishimura

Japan

Hiroshi Nishiura

Japan

Allan Nix

USA

Rosemary Nixon

Australia

Richard Njouom

Cameroon

James Nokes

Kenya

Robert Norton

Australia

Vas Novelli

UK

Norbert Nowotny

Austria

Saad Nseir

France

Georgia Ntani

UK

Maria Sofia Núncio

Portugal

Veronique Nussenblatt

USA

Balthazar Melchior Nyombi

Norway

Daniel O'Brien

Australia

Theresa Ochoa

Peru

Olga Ochoa-Gondar

Spain

Silvia Odolini

Italy

Darina O'Flanagan

Ireland

Dilara Ogunc

Turkey

Tinuade Ogunlesi

Nigeria

Olubunmi Ogunrin

Nigeria

Helen Oh

Singapore

Makoto Ohnishi

Japan

Shinichi Oka

Japan

Iruka Okeke

USA

Nwora Lance Okeke

USA

Lucy Okell

UK

Arinze Okoli

Norway

Ikechi Okpechi

South Africa

Claudia Oliveira

Brazil

Maura Oliveira

Brazil

Pablo Oliveira

France

Sigvard Olofsson

Sweden

Margaret Olsen

USA

Ashley Olson

UK

Geoffrey Omuse

Kenya

Bartholomew Ondigo

Kenya

Jason Ong

Australia

John Ong'Ech

Kenya

Clayton Onyango

Kenya

Eng Eong Ooi

Singapore

Eline Op De Coul

Netherlands

Steven Opal

USA

Joseph Oppong

USA

Faith Hope Among'In Osier

Kenya

Stanisław Ostrowski

Poland

Jose A Oteo

Spain

Jun Oto

Japan

Joanne O'Toole

Australia

Taketo Otsuka

Japan

Jean-Christophe Ouallet

France

Markus Pääkkönen

Finland

David Pace

Malta

John Paget

Netherlands

Madhukar Pai

Canada

Tibor Pál

United Arab Emirates

Domingo Palmero

Argentina

José Palomares

Spain

Mohan Pammi

USA

Mark Pandori

USA

Junxiong Pang

Singapore

George Papatheodoridis

Greece

Dimitrios pskevis

Greece

Subhash Parija

India

Saverio Parisi

Italy

Young Kil Park

South Korea

Philippe Parola

France

Giustino Parruti

Italy

Suzan Pas

Netherlands

Cesira Pasquarella

Italy

Pragna Patel

USA

Mical Paul

Israel

Christopher Peacock

Australia

Fiona Pearson

UK

Abraham Peedicayil

India

Trisha Peel

Australia

Trisha Peel

Australia

Camille Pelat

France

Ville Peltola

Finland

Yingwei Peng

Canada

Galo Peralta

Spain

Vera Lucia Pereira-Chioccola

Brazil

Santiago Perez Cachafeiro

Spain

Emilio Pérez-Trallero

Spain

David Perlin

USA

Guey Chuen Perng

Taiwan

Robert Perry

USA

Jonathan Peter

South Africa

Thomas Peterman

USA

Remco Peters

South Africa

Remco Peters

Netherlands

Tansy Peters

UK

Eskild Petersen

Denmark

Kyle Petersen

USA

Dennis Petrie

Australia

Linda Petrone

Italy

Nicola Petrosillo

Italy

Elisa Petruccioli

Italy

Salvatore Petta

Italy

Tung Phan

Bolivia

Julie Philley

USA

Aung Pyae Phyo

Thailand

Renata Picao

Brazil

Bruno Pichon

UK

Christine Pierce Campbell

USA

Antonio Pignatari

Brazil

Carlos Pigrau

Spain

Claudia Pileggi

Italy

Beth Piraino

USA

Lionel Piroth

France

Tyrone Pitt

UK

Paul Planet

USA

Jason Pogue

USA

Selda Polat

Turkey

Eva Polverino

Spain

Roberto Pontarolo

Brazil

Konstantinos Pontikis

Greece

Nurith Porat

Israel

Carolina Porras

USA

Maria Eugenia Portillo

Spain

Brunella Posteraro

Italy

Andrej Potthoff

Germany

Spyros Pournaras

Greece

Marinete Povoa

Brazil

Bruno Pozzetto

France

Therdsak Prammananan

Thailand

Rosa Prato

Italy

Ranjan Premaratna

Sri Lanka

Luiz Prestes-Carneiro

Brazil

Rebecca Prevots

USA

Erin Price

Australia

Kostas Priftis

Greece

Emilia Prospero

Italy

Roman Prymula

Czech Republic

Joan Puig-Barberà

Spain

Mirja Puolakkainen

Finland

Cheng-Feng Qin

China

Daniel Rabczenko

Poland

Dorthe Raben

Denmark

Andrea Rachow

Germany

Marie-Eve Raguenaud

Cambodia

Zeaur Rahim

Bangladesh

Ambak Kumar Rai

India

Kaitlin Rainwater Lovett

USA

Senaka Rajapakse

Sri Lanka

Jose Manuel Ramos

Spain

Dn Rao

India

J Rao

USA

Giammarco Raponi

Italy

Cristiane Rapparini

Brazil

Maria Raptopoulou-Gigi

Greece

Manoochehr Rasouli

Iran

Mohammad-Reza Rasouli

Iran

Mobeen Rathore

USA

Julian C Rayner

UK

Phillip Read

Australia

Andrew Redd

USA

Carrie Reed

USA

Mohamed Refai

Egypt

David Regan

Australia

Michael Reid

Botswana

Cavan Reilly

USA

Mitermayer Reis

Brazil

Richard Reithinger

USA

Bernhard Resch

Austria

Antonio Rezusta

Spain

Giovanni Rezza

Italy

Vanessa Ribeiro

Brazil

Josiah Rich

USA

Jan Hendrik Richardus

Netherlands

Mark Riddle

USA

Stefan Riedel

USA

Hans Rieder

Switzerland

Maria Helena Rigatto

Brazil

Leen Rigouts

Belgium

Eleanor Riley

UK

Felix C. Ringshausen

Germany

Andrew Riordan

UK

Giancarlo Ripabelli

Italy

David Ritchie

USA

Stephen Ritchie

New Zealand

Florence Robert-Gangneux

France

Jason Roberts

Australia

Roy Robins-Browne

Australia

Joan Robinson

Canada

Edward N. Robinson, Jr. Md Mph

USA

Jessica Robinson-Papp

USA

Juergen Rockstroh

Germany

Nils Rodhe

Sweden

Chaturaka Rodrigo

Sri Lanka

Marcelo Rodriguez Fermepin

Argentina

Jesús Rodríguez-Baño

Spain

Tom Rogers

UK

Stephen Rogerson

Australia

Petra Rohrbach

Canada

Emmanuel Roilides

Greece

Gustavo Romero

Brazil

Olivier Ronveaux

Burkina Faso

Ineke Rood

China

Carlos Ros

Switzerland

Marnie Rosenthal

USA

Alexander Rosewell

Papua New Guinea

Jonathan Ross

UK

John Wa Rossen

Netherlands

Klaus Rostgaard

Denmark

Kurt Roth

Germany

Katerina Roubalova

Czech Republic

Francois Rouet

Gabon

Djouaka Rousseau

Benin

Jason Roy

USA

Yuhua Ruan

China

Jeffrey Rubnitz

USA

Franco Maria Ruggeri

Italy

Markus Ruhnke

Germany

Mariagrazia Rumi

Italy

Maja Rupnik

Slovenia

Stefano Rusconi

Italy

Darren Russell

Australia

Kevin Russell

USA

Eva Ruzic-Sabljic

Slovenia

Claire Ryan

Myanmar

Alessandra Sacchi

Italy

David Sack

USA

S.M. Sadjjadi

Iran

Marco Safadi

Brazil

Mahboobeh Safaeian

USA

Amar Safdar

USA

Joseph Safdieh

USA

Kenneth Saffier

USA

Issaka Sagara

Mali

Evangelista Sagnelli

Italy

Evangelista Sagnelli

Italy

Seema Sahay

India

Edhyana Sahiratmadja

Indonesia

Kazuo Saita

Japan

Juan-Carlos Saiz

Spain

Nitin Saksena

Australia

Fernando Saldias

Chile

Claudiana Sales

Brazil

Cassandra Salgado

USA

Claudio Salgado

Brazil

Henrik Salje

USA

Marina Salvadori

Canada

Bernd Salzberger

Germany

Michael Samarkos

Greece

Vittorio Sambri

Italy

Moses Samje

Cameroon

David Samuels

USA

Uriel Sandkovsky

USA

Maria San-Martin

Spain

Maria Santoro

Italy

Sigrid Santos

Brazil

Bahador Sarkari

Iran

Bahador Sarkari

Iran

Michael Satlin

USA

Srinath Satyanarayana

Canada

Catherine Satzke

Australia

Andreas Sauerbrei

Germany

Paul Saunderson

Norway

Milton Saute

Israel

Hugo Sax

Switzerland

Dena Schanzer

Canada

Luciene Cardoso Scherer

Brazil

Mirte Scherpenisse

Netherlands

Maarten Franciscus Schim Van Der Loeff

Netherlands

Boris Valentijn Schmid

Netherlands

Herbert Schmitz

Germany

Thamarai Schneiders

UK

Peter Schubert

Canada

Philipp Schuetz

Switzerland

Eli Schwartz

Israel

Marin Schweizer

USA

Diana Scorpio

USA

Thomas Scriba

South Africa

Holly Seale

Australia

Patrick Sebastian

Germany

Eduardo Seclen

USA

Luigi Segagni Lusignani

Austria

Lorenzo Segovia

Mexico

Juliet Sekandi

USA

Masafumi Seki

Japan

Michael Selgrad

Germany

Liisa Selin

USA

Katja Seme

Slovenia

Joseph Sempa

Uganda

Arlene Sena

USA

Pascal Senn

Switzerland

Gunes Senol

Turkey

Mireille Serlie

Netherlands

Thea Sesardic

UK

Wai-Kay Seto

Hong Kong

Jane Seward

USA

Daniel Sexton

USA

Vivian Nguyan Shaahu

Nigeria

Leigh Anne Shafer

Canada

Dea Shahinas

Canada

Tengku Shahrul Anuar

Malaysia

Faiz Shakir

USA

Isdore Chola Shamputa

Canada

Shaobin Shang

USA

P Ravi Shankar

Nepal

Eugene Shapiro

USA

Kathleen Shapley-Quinn

USA

Batu Sharma

USA

Marie-Anne Shaw

UK

Johnathan Sheele

USA

Samuel Shelburne

USA

Aimee Shen

USA

Liping Shen

China

Ji-Fang Sheng

China

Wang-Huei Sheng

Taiwan

Lin Shi

China

Weifeng Shi

China

Bruce Shiramizu

USA

Mitrakrishnan Shivanthan

Sri Lanka

Shmuel Shoham

USA

Sunao Shoji

Japan

Anna Shore

Ireland

Nabin Shrestha

USA

Farideh Siavoshi

Iran

Marco Siccardi

UK

Mario Sideri

Italy

Hanna Sidjabat

Australia

Mark Siedner

USA

Yardena Siegman-Igra

Israel

Kim Sigaloff

Netherlands

Tassanee Silawan

Thailand

Benjamin Silk

USA

Aristeu Silva

Brazil

Denise Silva

Brazil

Jacobo Silva

Spain

Umberto Simeoni

France

Fernando Simón

Spain

Leonardo Simonella

Singapore

Prof Sarman Singh

India

Edford Sinkala

Zambia

Jean-Marie Sire

France

Charalambos Siristatidis

Greece

Leung Kei Siu

Taiwan

Anders Sjostedt

Sweden

Rod Skinner

UK

Svetoslav Slavov

Brazil

Rachel Slayton

USA

Jihad Slim

USA

Robert Slinger

Canada

Pierre Smeesters

Belgium

Vitaly Smelov

France

Amber Smith

USA

Anthony Smith

South Africa

David Smith

USA

David Smith

Australia

Edward Smith

UK

Elaine Smith

USA

Kirsty Smith

Australia

Tara Smith

USA

Alison Smith-Palmer

UK

Teemu Smura

Finland

Davida Smyth

USA

Mette Søgaard

Denmark

Kate Soldan

UK

Alicia Solorzano

USA

Geetika Sood

USA

Nieves Sopena

Spain

Heng Sopheab

Cambodia

Alessandro Soria

Italy

Giovanni Sotgiu

Italy

Sara Soto

Colombia

John Spinelli

Canada

Bruno Spire

France

Philip Spradling

USA

Nicola Squillace

Italy

Chandrashekhar Sreeramareddy

Nepal

Sanjeeva Srivastava

India

Gaby Sroczynski

Austria

Simona Stager

Canada

Cecilia Stålsby Lundborg

Sweden

Gerold Stanek

Austria

Jeffrey Starke

USA

Marc Steben

Canada

Ines Steffens

Sweden

Catherine Stein

USA

Mart Stein

Netherlands

Martin Steinau

USA

Florian Steiner

Germany

Robert Steinglass

USA

Jochen Steinmann

Germany

David Stephens

USA

Goran Stevanovic

Serbia

Vanessa Stevens

USA

Joanna Stewart

New Zealand

Ellen Stobberingh

Netherlands

Mark Stoove

Australia

Valentina Stosor

USA

Jason Stout

USA

Norval Strachan

UK

Raphael Stricker

USA

Robert Striker

USA

Christina Strube

Germany

James Stuart

UK

Lieven Stuyver

Belgium

Chien-Wei Su

Taiwan

Wei-Juin Su

Taiwan

Jose Suaya

USA

Toshiro Sugimoto

Japan

Pär-Daniel Sundvall

Sweden

Xun Suo

China

Michael Surette

Canada

Troy Sutton

USA

Yoshiyuki Suzuki

Japan

Youichi Suzuki

Singapore

Sathyamangalam Swaminathan

India

Faisal Syed

USA

Jean-Luc Szpakowski

USA

Frank Tacke

Germany

Tomohiko Takasaki

Japan

Howard Takiff

Venezuela

Paul Tambyah

Singapore

Cheong Huat Tan

Singapore

Kevin Shyong-Wei Tan

Singapore

Lionel Tan

UK

Thuan-Tong Tan

Singapore

Yi Tan

USA

Yasuhito Tanaka

Japan

Juhi Taneja

India

Julian Wei Tze Tang

Singapore

Jamshid Tanha

Canada

Kiyosu Taniguchi

Japan

Julian Tanner

Hong Kong

Panayotis T. Tassios

Greece

Alper Tekeli

Turkey

Laura Temime

France

Edamisan Temiye

Nigeria

David Templeton

Australia

Daniel Tena

Spain

Christine Teng

Singapore

Hwa-Jen Teng

Taiwan

Jeanette Teo

Singapore

Eyasu Teshale

USA

Khoa Thai

Netherlands

Anne Thebault

France

Nicole Theodoropoulos

USA

Vincent Thibault

France

Robert Thimme

Germany

Mark Thomas

New Zealand

Kimberly Thompson

USA

Reimar W Thomsen

Denmark

Koh Cheng Thoon

Singapore

Bruce Thorley

Australia

Kamala Thriemer

Belgium

Franck Thuny

France

Guy Thwaites

UK

Thorsten Thye

Germany

Zhang Ting

China

Kelvin To

Hong Kong

Catherine Todd

USA

Rafal Tokarz

USA

Steven Tong

Australia

Winnie Tong

Australia

Zeynep Topkarci

Turkey

Nuria Torner

Spain

Kwasi Torpey

Nigeria

Sherry Towers

USA

Mehlika Toy

USA

Howard Trachtman

USA

Francesco Trapasso

Italy

Christopher Troeger

USA

Annet Troelstra

Netherlands

Jih-Jin Tsai

Taiwan

Sarah Tschudin Sutter

Switzerland

Lekha Tuli

India

Elaine Tuomanen

USA

Katy Turner

UK

Robin Turner

Australia

Jane Turton

UK

Jimmy Twin

Australia

Mark Tyndall

Canada

Greg Tyrrell

Canada

Edet Udo

Kuwait

Kerry Uebel

South Africa

Julia Uhanova

Canada

James Uhl

USA

Anne-Catrin Uhlemann

USA

Christian Ukuru

Nigeria

Sebastian Ulbert

Germany

Meera Unnikrishnan

UK

Vytautas Usonis

Lithuania

Riccardo Utili

Italy

Anneli Uuskula

Estonia

Pieter Uys

South Africa

Antti Vaheri

Finland

Zoltan Vajo

Hungary

Francesca Valent

Italy

Louis Valiquette

Canada

Olaf Valverde Mordt

Switzerland

Liselotte Van Asten

Netherlands

Françoise Van Bambeke

Belgium

Alje Van Dam

Netherlands

David Van De Vijver

Netherlands

Janneke Van De Wijgert

Netherlands

Annemiek Van Der Eijk

Netherlands

Lia Van Der Hoek

Netherlands

Wim Van Der Hoek

Netherlands

Mark Van Der Linden

Germany

Barbara Van Der Pol

USA

Marianne A.B. Van Der Sande

Netherlands

A.M. Van Furth

Netherlands

Perry Van Genderen

Netherlands

Albert Jan Van Hoek

UK

Jakko Van Ingen

Netherlands

Frank Van Leth

Netherlands

Wilfrid Van Pelt

Netherlands

Annelies Van Rie

USA

Carla Van Tienen

Gambia

Michele Van Vugt

Netherlands

Nicholas Van Wagoner

USA

Carl Van Walraven

Canada

Gijs Th. J. Van Well

Netherlands

Russell Vance

USA

Anne-Mieke Vandamme

Bermuda

Anne-Mieke Vandamme

Belgium

Wannes Vanderhaeghen

Belgium

Olli Vapalahti

Finland

Luca Varani

Switzerland

Konstantinos Vardakas

Greece

Anne Vardo-Zalik

USA

Stephen Vaughan

Canada

Ravindra Vegunta

USA

Thirumalaisamy Velavan

Germany

Irene Veldhuijzen

Netherlands

Aristea Velegraki

Greece

Inga Velicko

Sweden

Akke Vellinga

Ireland

Valdilea Veloso

Brazil

Pradhib Venkatesan

UK

Arjun Venkatesh

USA

Marietjie Venter

South Africa

Sandro Vento

Botswana

Henri Verbrugh

Netherlands

Theo Verheij

Netherlands

Rita Verhelst

Belgium

Chris Verhofstede

Belgium

Rajesh Verma

India

Johannes Vermehren

Germany

Ej Verschoor

Netherlands

Hans Verstraelen

Belgium

Jaco Verweij

Netherlands

Timo Vesikari

Finland

Cecile Viboud

USA

Peter Vickerman

UK

Francesc Vidal

Spain

Marjorie Vieira Batista

Brazil

Livia Villar

Brazil

Paolo Villari

Italy

George Viola

USA

Rodolfo Viotti

Argentina

Eric Viscogliosi

France

Astra Vitkauskiene

Lithuania

Miguel Viveiros

Portugal

Erika Vlieghe

Belgium

Lenka Vodstrcil

Australia

Martin Vogel

Germany

Liffert Vogt

Netherlands

Antonio Volpi

Italy

Heike Von Baum

Germany

Andrea Von Groll

Brazil

Lorenz Von Seidlein

Australia

Ralf-Peter Vonberg

Germany

John Vulule

Kenya

Kamenna Vutova

Bulgaria

Indra Vythilingam

Malaysia

William Wade

UK

Hans-Joachim Wagner

Germany

Stephen Wagner

USA

Catriona Waitt

Malawi

David Walker

USA

Jennifer Walker

Australia

Jacco Wallinga

Netherlands

Robert Wallis

USA

Martine Wallon

France

Jann-Yuan Wang

Taiwan

Jiyao Wang

China

Ming Wang

China

Yue Wang

China

David Wareham

UK

Justin Waring

Australia

Toru Watanabe

Japan

Emma Webb

UK

Richard Webby

USA

Joerg Weber

Germany

Scott Weese

Canada

David Weetman

UK

Nina Weis

Denmark

Martijn Weisfelt

Netherlands

Laurence Weiss

France

Maja Weisser

Switzerland

Christian Wejse

Denmark

Susan Welburn

UK

Dirk Werber

Germany

Jim Werngren

Sweden

Wayde Weston

USA

David Whiley

Australia

Denise Whitby

USA

Richard White

UK

Andrew Christopher Whitelaw

South Africa

Douglas Widman

USA

Nathan Wiederhold

USA

Kerrie Wiley

Australia

Katalin Wilkinson

South Africa

Julie Wilson

UK

Lauren Wilson

USA

Peter Wilson

UK

Samuel Wilson

USA

Kevin Winthrop

USA

Xavier Wittebole

Belgium

Camilla Wiuff

UK

Martin Wolkewitz

Germany

Karen Wong

USA

Kum Thong Wong

Malaysia

James Wood

Australia

Andrew Woodhouse

UK

Charles Woodrow

Thailand

Dan Wootton

UK

Gary Wormser

USA

William Worodria

Uganda

Stephen Wright

UK

Ho-Sheng Wu

Taiwan

Jeng-Yuan Wu

Taiwan

Lii Tzu Wu

Taiwan

Ting Wu

China

Peter Wutzler

Germany

Jianchun Xiao

USA

Yanni Xiao

China

Yanyu Xiao

Canada

Cuiling Xu

China

Howard Xu

USA

Jiancheng Xu

China

Laith Yakob

Australia

Javed Yakoob

Pakistan

Walelegn Worku Yallew

Ethiopia

Yan Yana

China

Pan-Chyr Yang

Taiwan

Kaihu Yao

China

Maosheng Yao

China

Trai-Ming Yeh

Taiwan

Jung Sook Yeom

South Korea

Sabine Yerly

Taiwan

Panayiotis Yiallouros

Cyprus

Solomon A Yimer

Norway

Guo Rong Yin

China

Louis-Marie Yindom

UK

Cyril Yip

Hong Kong

Jean Cyr Yombi

Belgium

Junichi Yoshida

Japan

Hisao Yoshikawa

Japan

Edward Young

USA

Jo-Anne Young

USA

Linda Young

USA

Fangyou Yu

China

Wen-Liang Yu

Tajikistan

Yunsong Yu

China

Yuhong Yuan

Canada

Man-Fung Yuen

Hong Kong

Heather Yun

USA

Nadezhda Yun

USA

Chee-Fu Yung

Singapore

Mohd Imran Yusof

Malaysia

Kamran Yusuf

Canada

Jean Ralph Zahar

France

Janine Zahlten

Germany

Dario Zamboni

Brazil

Olympia Zarkotou

Greece

Alexandre Zavascki

Brazil

Jonathan Zelner

USA

Bei-Bei Zhang

China

Chiyu Zhang

China

Gong Zhang

China

Lei Zhang

Australia

Longxian Zhang

China

Lu Zhang

China

Tuohong Zhang

China

Wenbao Zhang

China

Zongde Zhang

China

Naiqing Zhao

China

Xf Zheng

China

Changtai Zhu

China

Fengcai Zhu

China

Wei Zhu

China

Panayiotis Ziakas

USA

Marya Zilberberg

USA

Thomas Zoller

Germany

Stefano Zona

Italy

Huachun Zou

Australia

Hosam Zowawi

Australia

Ines Zulantay

Chile

Axel Zur Hausen

Netherlands

## Pre-publication history

The pre-publication history for this paper can be accessed here:

http://www.biomedcentral.com/1471-2334/14/20/prepub

